# Supplementary material for: AeroPath: An airway segmentation benchmark dataset with challenging pathology and baseline method
Source: PLoS One. 2024 Oct 2;19(10):e0311416. doi: 10.1371/journal.pone.0311416 (PMC11446458; doi:10.1371/journal.pone.0311416)
Supplement: Attachment — Submitted filename: ResponseToReviewers-AeroPath-PLOSONE-Aug2024.pdf [file pone.0311416.s001.pdf]

Dear Peng Geng, Academic Editor of PLOS ONE,

Many thanks for the positive feedback on our work, and I hope the updated manuscript is clearer and more informative. We think that your suggestions have enhanced the presentation of the work. Below, you find our point-by-point response to the reviewers' comments.

Reviewers' comments:

Reviewer #1:
This paper employs a robust methodology for training and validating the AGU-Net architecture using a 5-fold cross-validation approach on the ATM'22 dataset. The authors benchmark their proposed method against multiple established baseline methods, including FAST, BronchiNet, TotalSegmentator, and MEDSeg, which provides a comprehensive evaluation of performance metrics.

This paper employed a 5-fold cross-validation approach for training and validating their models, which is a standard practice in machine learning to ensure that the model's performance is robust and not overly fitted to a specific subset of the data. It also utilized multiple performance metrics, including Dice Similarity Coefficient (DSC), precision, sensitivity, and specificity, to evaluate the segmentation results against various baseline methods.

The AeroPath dataset, which includes the annotated CT scans used in their study, and the best trained model are openly accessible.

Thank you for the positive response.

Reviewer #2:
This manuscript contains two contributions to the analysis of CT images of the lungs in order to support early diagnosis and improved treatment. The first is a description of a new benchmark dataset with 27 cases of more severe pathologies than available in existing datasets. The second is a description of a multiscale fusion design for automated airway segmentation. Only the first contribution is mentioned in the title of the paper which is somewhat misleading. I suggest the title is modified to cover both parts of the paper. Also in the text of the paper the description of the dataset and the developed segmentation model is very much integrated. The paper could become more clear if this was more separated.

We agree with the comment and adjusted the title as follows:

> *"AeroPath: An airway segmentation benchmark dataset with challenging pathology <u>and baseline method"</u>*
> *In addition, we have restructured the Materials and Methods section to further highlight our two contributions over the proposed dataset and segmentation method. Together with the newly added figure portraying the architecture design, we believe the section to be clearer to the reader.*

The paper is well written and I only have a few detailed comments and suggestions:
In the description of data augmentation it is stated that a number of transforms were applied to each input sample with a probability of 50%. But several of the transforms are not binary but rather includes a choice of rotation in the range +20 to +20 degrees, translation up to 20% of the axis and zoom in the range 50 through 150%. They can not be chosen with 50% probability. Please clarify how these augmentations were chosen. Also were all kinds of augmentations used for all samples?

> *We have revised the phrasing in the manuscript to make it clearer to the reader. Each data augmentation method had a probability of 50% to be applied for any new input sample, in a consecutive fashion. For the randomly selected data augmentation techniques, a random value inside the described ranges was then picked.*

It is described in the section on validation studies that the developed method could not be compared to the five best performing methods from the ATM22 challenge due to lack of code or trained models availability. In the abstract it is stated that the developed model was evaluated against competitive open source methods. It would be fair to note there the problem with access to compare against the best methods or at least to discuss this issue further.

> *The topic has been further addressed in the Discussion section. We do agree that a comparison to the ATM22 methods would have been an interesting inclusion into the article but unfortunately, we do not have any control over the accessibility of other methods. However, we believe to have used strong state-of-the-art methods to compare our method against.*

In the evaluation of methods performance two custom defined measures are used TD and BD. The meanings of those terms are briefly described in the Metric section but the definition could be made a bit more mathematically well defined. Also in the Tables 2 and 3 the terms TD and BD (and DSC) are used without explanation, making it necessary to read the text to understand the tables. It would make the paper easier to read if the definitions were included in the table legends.

> *We have added the mathematical equation as suggested and also expanded the Table legend to make it easier to read.*

It is stated that the ground truth annotations were partly based on FAST+BronchiNet while FAST+BronchiNet is one of the methods being compared in the tables. Please comment about how or to what extent this may bias the comparisons results.

> *We agree with the reviewer and have included text clearifying this in the Discussion section.*
